# Royal jelly a promising therapeutic intervention and functional food supplement: A systematic review

**DOI:** 10.1016/j.heliyon.2024.e37138

**Published:** 2024-08-30

**Authors:** Rajesh Kumar, Ankita Thakur, Suresh Kumar, Younis Ahmad Hajam

**Affiliations:** a Department Biosciences, Himachal University, Shimla, Himachal Pradesh-171005, India; b Department of Life Sciences and Allied Health Sciences, Sant Baba Bhag Singh University, Jalandhar, Punjab -144030, India

**Keywords:** Apitherapy, Functional food, Pharmacological activities, Complementary medicine

## Abstract

Royal jelly (RJ), a secretion produced by honeybees, has garnered significant interest for its potential as a therapeutic intervention and functional food supplement. This systematic review aims to synthesize current research on the health benefits, bioactive components, and mechanisms of action of RJ. Comprehensive literature searches were conducted across multiple databases, including PubMed, Scopus, and Web of Science, focusing on studies published from 2000 to 2024 (April). Findings indicate that RJ exhibits a wide range of pharmacological activities, including anti-inflammatory, antioxidant, antimicrobial, and anti-aging effects. Beneficial biological properties of RJ might be due to the presence of flavonoids proteins, peptides, fatty acids. Both preclinical and clinical studies have reported that RJ improves the immune function such as wound healing, and also decreases the severity of chronic diseases including diabetes and cardiovascular disorders. The molecular mechanisms underlying these effects involve modulation of signalling pathways such as NF-κB, MAPK, and AMPK. Despite promising results, the review identifies several gaps in the current knowledge, including the need for standardized dosing regimens and long-term safety assessments. Furthermore, variations in RJ composition due to geographic and botanical factors necessitate more rigorous quality control measures. This review underscores the potential of RJ as a multifunctional therapeutic agent and highlights the necessity for further well designed studies to fully elucidate its health benefits and optimize its use as a functional food supplement.

## Highlights

• Royal jelly is rich in proteins, vitamins, minerals, and bioactive compounds.
• Royal jelly has nutritional value and protects against cellular damage.
• Royal jelly has anti-inflammatory properties, antimicrobial, and metabolic regulator.
• Royal jelly boosts the functional ability of the immune system.
• Royal jelly is used for anticancer, wound healing, antiaging, and neuroprotection.

## Graphical abstract

**Figure:**
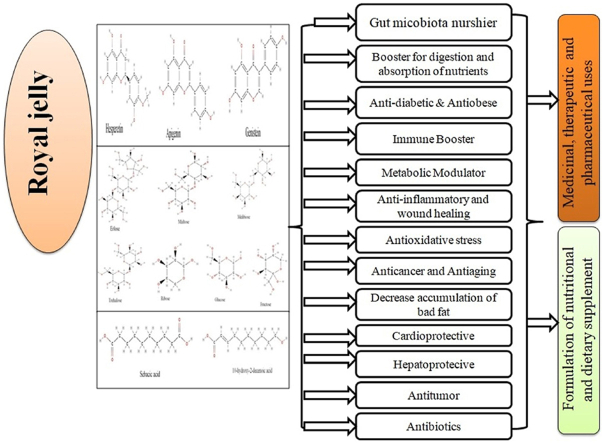

## 1 Introduction

Honey bees are eusocial insects, holding special status in nature by providing various ecosystem services such as pollination, environmental indicators, production of honey, and other valuable products such as propolis, wax, RJ, etc. Among these products, honey is perhaps the most widely known and most widely used product. RJ, a milky secretion produced by worker bees 1–3 days old larvae of all the honeybee castes (queen, worker, and drone) are fed with RJ. Well known for its purported health benefits, RJ has garnered considerable attention in scientific research and traditional medicine alike. Bee pollen, another product collected by honey bees, is a nutrient rich substance gathered from flowers and used as a food source for the hive. Rich in vitamins, minerals, proteins, and antioxidants, bee pollen has been hailed for its potential health-promoting properties and is consumed by humans in various forms, including supplements and health foods (Table 1). Propolis, often referred to as “bee glue," is a resinous substance collected by bees from tree buds and sap. Utilized by bees to seal cracks in the hive and defend against pathogens, propolis exhibits antimicrobial, anti-inflammatory, and antioxidant properties, making it a subject of interest in both traditional and modern medicine. Bee venom, produced by specialized glands in the abdomen of worker bees, contains a complex mixture of peptides and enzymes with diverse biological activities. While bee stings can elicit painful reactions in humans, controlled exposure to bee venom has been explored for its potential therapeutic effects in conditions ranging from arthritis to certain types of cancer [1,2]. In this scientific exploration of RJ has been uncovered in order to explore its potential benefits for human health and well-being, underscoring the profound interconnectedness between humans and the natural world.

**Table 1:** Summarizes the composition, bioactive compounds, and health benefits associated with various bee products, providing a concise overview of their medicinal properties.

Bee Product	Composition	Bioactive Compounds	Health Benefits
Honey	Carbohydrates (glucose, fructose), di- and oligosaccharides, organic acids, enzymes, vitamins, amino acids, peptides	Polyphenols, flavonoids	Anti-inflammatory, antioxidant, potential prevention of diseases like cancer, diabetes, obesity
Propolis	Resin, wax, essential oils, pollen, esters, diterpenes, lignans, alcohols, vitamins, flavonoids, amino acids, fatty acids, minerals	Caffeic acid phenethyl ester (CAPE), flavonoids, phenolic acids	Neuroprotective effects, antioxidative, anti-cancer properties
Bee Pollen/Bee Bread	Carbohydrates, proteins, vitamins, amino acids, lipids, fatty acids	Phenolic compounds	Health-promoting activities, antimicrobial, anti-inflammatory
Bee Venom	Carbohydrates, lipids, proteins, enzymes (hyaluronidase, phospholipase A2), peptides (melittin, apamin, MCD), pheromones, minerals	Phospholipase A2, hyaluronidase, peptides	Anti-inflammatory, immune system modulation, potential neuroprotective and anti-cancer properties
Royal Jelly	Sugars, lipids, proteins, amino acids, vitamins, minerals	Royalactin, hydroxy-decenoic acid (10-HDA), proteins	Potential health maintenance, pharmaceutical applications

## 2 Urgency and need of this review

The urgency of this review arises from the growing interest in the therapeutic effects of RJ, a natural substance produced by worker honeybees. Despite its traditional use and anecdotal health benefits, scientific understanding of its mechanisms and clinical applications remains limited. Given the increasing demand for alternative therapies and potential healthcare innovations, a comprehensive critical appraisal is essential. This review aims to analyze existing evidence, elucidate the pharmacological effects of RJ, and offer insights into its therapeutic potential. Through rigorous analysis, it seeks to fill research gaps, to guide future research, and potentially unlock new therapeutic interventions. This appraisal will help distinguish evidence-based therapeutic potential from conjecture, thereby enriching our understanding of natural remedies and potentially paving the way for novel healthcare innovations.

## 3 Royal jelly

RJ, produced by worker bees from the hypopharyngeal and mandibular glands through partial digestion of honeydew, is crucial for the development and caste differentiation of honeybee larvae [3,4]. 1-3 days-old larvae of all the honeybee castes (queen, worker, and drone) are fed with RJ. RJ is a yellowish-white gelatinous substance made from proteins, carbohydrates, lipids, and vitamins produced by secretory cells in the glandular acini [5,6]. It is transported to the mouthparts of worker bees and consumed by the queen and larvae, supporting colony growth.

Historically significant in ancient Greek and Egyptian cultures, RJ was linked to immortality and beauty, notably used by Cleopatra. Since the 1960s, research in apitherapy has explored its health benefits, including antimicrobial, anti-aging, anti-tumor, antioxidative, anti-diabetic, immunomodulatory, and neuroprotective effects [[6], [7], [8], [9]]. These properties suggest potential applications for conditions like infertility, digestive disorders, Alzheimer's, and depression [10,11].

**Fig. 1:**
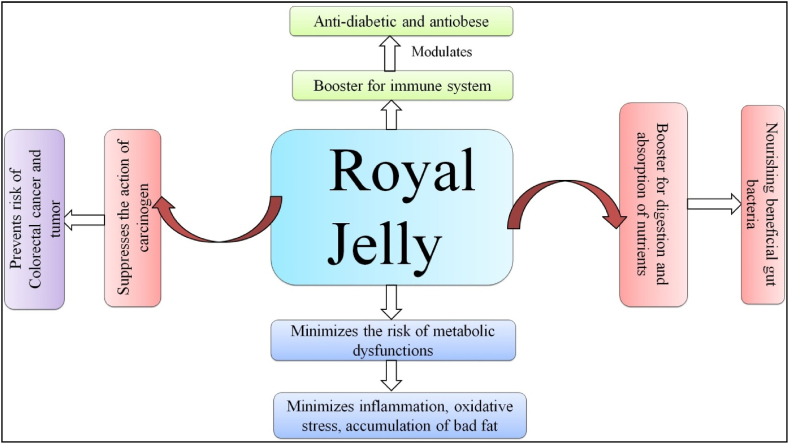
Showing the role of royal jelly (RJ) in the management of different disease.

In Chinese culture, RJ is widely used as a dietary supplement and cosmetic ingredient, offering benefits such as maintaining reproductive health, enhancing memory, preventing dementia, and reducing anxiety. Its anti-inflammatory action helps intestinal health by modulating cytokine levels (Fig. 1). The impact of RJ on fertility may be due to its ability to increase hormone production, and its anti-aging properties position it as a promising ingredient in medicinal and cosmetic formulations (Table 2). The longevity-promoting effects in queen bees, attributed to Major RJ Proteins (MRJPs), suggest potential implications for human lifespan extension (Table 2). Proper handling and cold storage are essential due to its perishable nature. RJ regulates different physiological functions in bees and indicates potential regulatory roles in humans, making it relevant for commercial, cosmetic, and medicinal applications (Table 3). Furthermore, its rich nutritional composition, comprising proteins, vitamins, minerals, and unique bioactive compounds, underscores its therapeutic potential. The enduring fascination with RJ in traditional medicine underscores its enduring legacy as a natural remedy with profound implications for human health and wellness (Table 3).

**Table 2:** Shows the physical characteristics of royal jelly (RJ), health benefits, major proteins, lipid, carbohydrate, Other Components, Storage Recommendations, Importance for Bees and Pharmacological Focus content.

Component	Description
Appearance	White or yellowish gelatinous substance
Taste	Sweet-sour
pH	3.4–4.5
Main Health Benefits	Antioxidant, Anti-inflammatory, Neurotrophic, Hypotensive
	Antidiabetic, Antihypercholesterolemic, Antirheumatic Antitumor
	Antifatigue, Antimicrobial, Nematocidal, Anti-aging

**Component**	**Description**	**Pharmacological Importance**
Major Proteins	- Major royal jelly proteins (MRJPs) (constitute 50 % of dry matter weight) Royalisin Jelleines	MRJPs: Active ingredients<br>− Antioxidative peptides (up to 29 identified)
	Aspimin<br>− Newly discovered proteins	
Lipid Fraction	- 3–6% of wet weight	- Trans-10-hydroxy-2-decenoic acid (10-HDA): Exhibits various biological properties (anti-aging, neurogenic, anticancer, antiobesity, antibacterial)
	7–18 % of dry weight	
	Mainly short hydroxyl fatty acids (constitute 80–85 %)	
	Also contains phenols (4–10 %), waxes (5–6%), steroids (3–4%), phospholipids (0.4–0.8 %)	
Carbohydrates	− 90 % fructose and glucose 7.5–16 % of RJ	
Other Components	Vitamins, Minerals, Phenols, Esters, Aldehydes, Ketones	
	Alcohol, Bioactive substances” like ACh and nucleotides	
Storage Recommendations	- Should be stored frozen to retain biological properties	
	Storage above 5 °C reduces soluble nitrogen and free amino acids	
Importance for Bees	- Sole food for bee queens throughout their lifespan Associated with bee queen's “long lifespan, high fertility, and excellent learning and memory ability”	
Pharmacological Focus	Investigation of anti-aging properties with focus on cognitive function in advanced aging and Alzheimer's disease (AD)	
	Reviewing studies on RJ's effects “on cognitive aging and AD pathology in cell cultures, animal models, and” humans when possible	
	Elaborating on molecular changes underlying these effects

**Table 3:** This table provides a summary of the various therapeutic implications of royal jelly, highlighting its potential benefits for overall health and well-being.

Therapeutic Implication	Description
Antioxidant Properties	Flavonoids, phenolic compounds, and vitamins help to neutralize free radicals and reduce oxidative stress.
Anti-inflammatory Effects	Components such as “10-Hydroxy-2-decenoic acid (10-HDA)” exhibit anti-inflammatory properties, and reduce inflammation in arthritis and skin irritations.
Immune System Support	Boosts the immune system by stimulating the production of immune cells such as lymphocytes and macrophages, thus improving overall immune function and response to infections.
Wound Healing	The presence of proteins, vitamins, and amino acids in royal jelly can accelerate wound healing by promoting tissue repair and regeneration, making it beneficial for treating cuts, burns, and other skin injuries.
Cardiovascular Health	Royal jelly contains compounds like fatty acids and peptides that may have cardio-protective effects by lowering cholesterol, improving blood vessel function, and reducing the risk of cardiovascular diseases.
Neuroprotective Effects	Compounds like 10-HDA and royalisin have neuroprotective properties, potentially enhancing the survival of nerve cells in conditions like Alzheimer's, Parkinson's, and cognitive reduction.
Anti-diabetic Properties	Royal jelly may be a potential adjunctive therapy for managing diabetes by regulating blood sugar levels and improving insulin sensitivity.
Anti-cancer Potential	Royal jelly has been found to have anti-cancer properties through mechanisms like inhibiting tumor cell growth, inducing apoptosis, and enhancing the body's immune response against cancer cells.
Hormonal Balance	Royal jelly contains hormone-like substances such as royalactin, which may help regulate hormonal balance, particularly in reproductive health, by supporting fertility, and menstrual regularity, and relieving symptoms of hormonal imbalances such as PMS and menopause.
Skin Health and Beauty	The vitamins, minerals, and amino acids present in royal jelly contribute to skin nourishment and rejuvenation, promoting a healthy complexion, reducing signs of aging like wrinkles and fine lines, and alleviating skin conditions such as eczema and acne.

## 4 Methods

The systematic review of randomized controlled trails (RCTs) was carried out following the rules and procedure mentioned in the Cochrane Handbook for systemic review analysis [12].

### 4.1 Search strategy

A comprehensive computerized search was conducted across different databases such as PubMed (http://www.ncbi.nlm.nih.gov/pubmed), Web of Science (https://mjl.clarivate.com/search results), Science Direct (https://www.sciencedirect.com/), and Google Scholar (https://scholar.google.com/) 2000 to 2024. Moreover, we carefully reviewed the reference lists of all significant studies and reviews. The following terms were used either singly or in combination as inclusion criteria: “anti-inflammatory," “antibacterial," “anti diabetic," “apoptotic," “respiratory," “gastrointestinal," “cardiovascular," and “nervous system," neurodegenerative diseases (e.g., Alzheimer's, Parkinson's), hepatotoxicity, renal toxicity, metabolic disorders (e.g., diabetes, obesity, hyperlipidaemia), reproductive disorders (e.g., PCOS, infertility and oligospermia), and viral diseases (e.g., COVID-19). After about 200 results were found, their abstracts were examined to determine their applicability. About 110 research and review publications were carefully scrutinized in order to compile the current article, with additional exclusion criteria such non-English language and lack of full-text manuscript availability being applied.

### 4.2 Study selection

**Fig. 2:**
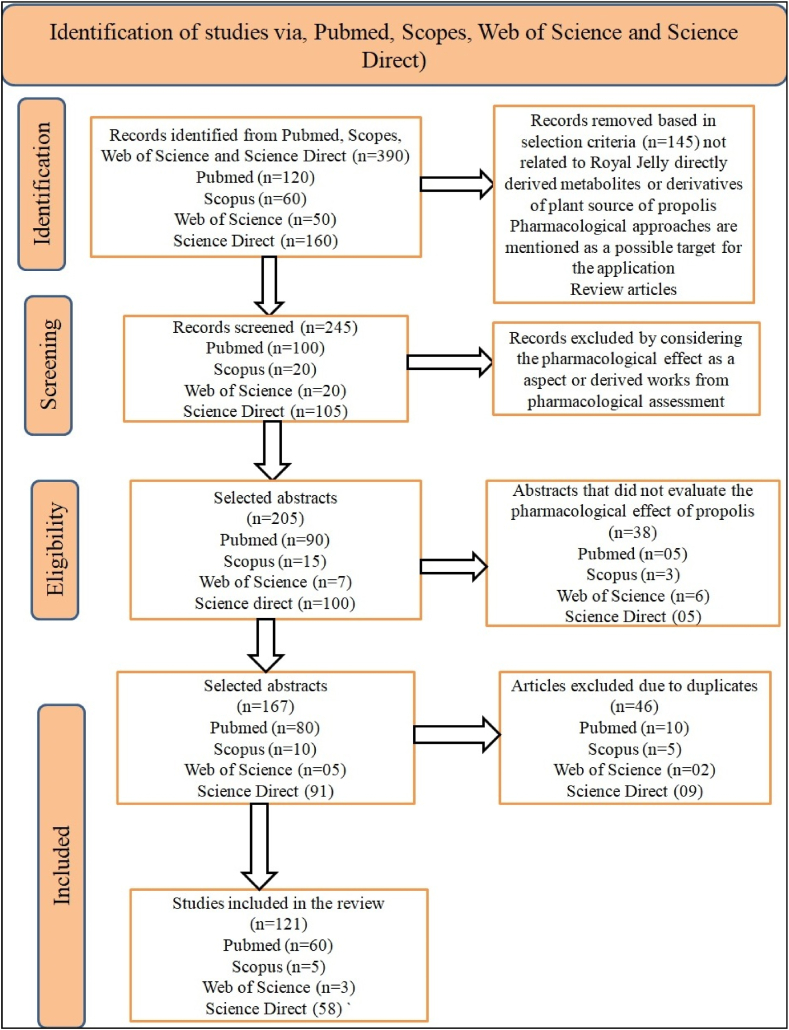
Flow chart of the process of the study selection.

Two authors independently screened titles and abstracts having relevance with trials, followed by retrieval and examination of the full length text of the paper to screen out the relevant trial studies having relevance with our review article. Relevant data were extracted from all these articles and summarized by applying the standardized format to evaluate the quality of study and to synthesis evidence. The extracted data encompassed information like the title, name of authors, year of publication, and name of country, parameters of the studied trail including biological, pharmacological and food supplement roles of RJ. The systematic review and meta-analysis did not include any studies that used RJ as a medicinal solution for a variety of disorders with treatment durations shorter than one week. Every stage of the study selection procedure was carried out separately by at least two authors to assure accuracy, and disagreements were settled by consensus (Fig. 2).

### 4.3 Data extraction

Data abstraction form was used to individually and twice extract pertinent data from each included article. Dispute resolution was accomplished through consultation. The following details were recorded: the type and dosage of RJ, the type of placebo or control group, the duration of the treatment, the formulation used, the minimal active concentration tested, the duration of the study, the model used (in vitro or *in vivo* study), and other basic pharmacological data; the level of interest outcomes (pharmacological properties such as antidiabetic, anticancer, cardiovascular, antihypertensive, anti-inflammatory, immunomodulatory and reproductive related studies).

## 5 Chemical composition of RJ

RJ is an acidic secretion with a pH of 3.5–4.2. It mostly consists of water 60–70 %, sugars 7–18 %, proteins 9–18 %, lipids 3–8%, minerals, and trace amounts of vitamins. Major lipids in it consist of 10-hydroxy-2-decanoic acid and sebacic acid [13]. 10H2DA is known for its anti-cancerous and anti-angiogenic activity whereas sebacic acid has anti-aging effects. Major RJ Proteins (MRJPs) present in the RJ increases the lifespan of Queenbee (Table 4). It consists of peptides like royalisin, jelleines and royalactina. The various pharmacological aspects of RJ are attributed to its unique and rich composition of proteins, carbohydrates, vitamins, lipids, minerals, flavonoids, and polyphenols along with various biologically active substances (Table 5).

**Table 4:** Gross chemical composition of royal jelly [14].

Chemical Component	Percentage (%)
Water	60–70
Proteins	10–18
Carbohydrates	11–23
Lipids (Fats)	3–8
Ash	1–2
Vitamins	Trace amounts
Minerals	Trace amounts
Enzymes	Trace amounts
Hormones	Trace amounts
Other Bioactive Compounds	Trace amounts

**Table 5:** Phytochemical profile of royal jelly.

Bioactive Compound	Percentage (%)
Royalactin	1–3
10-Hydroxy-2-decenoic acid (10-HDA)	1–3
Acetylcholine	0.5–1
Adenosine	0.2-0.5
Nucleotides (AMP, ADP, ATP)	0.1-0.3
Gamma-aminobutyric acid (GABA)	0.1-0.2
Polyphenols	0.1-0.2
Flavonoids	Trace amounts
Phospholipids	Trace amounts
Sterols	Trace amounts
Growth factors (EGF and TGF)	Trace amounts

### 5.1 Carbohydrates

**Fig. 3:**
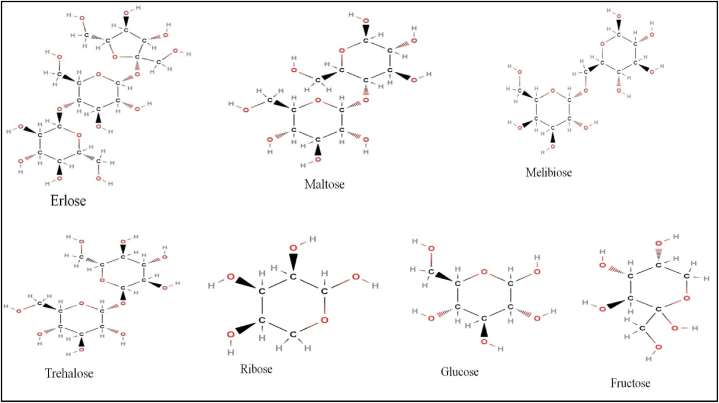
Showing 2D chemical structures of different carbohydrates present in the royal jelly.

RJ contains approximately 7.5–15 % sugars, with fructose and glucose comprising the majority, making up around 90 % of the sugar content. Additionally, maltose, trehalose, melibiose, ribose, and erlose constitute about 0.8–3.6 % of the sugar composition in RJ [15] (Fig. 3). These sugars present in RJ act as aphago stimulants, operating through insulin signalling cascades and nutrient sensing pathways, thereby enhancing nutrient intake crucial for larval and queen development [16].

### 5.2 Lipids

**Fig. 4:**
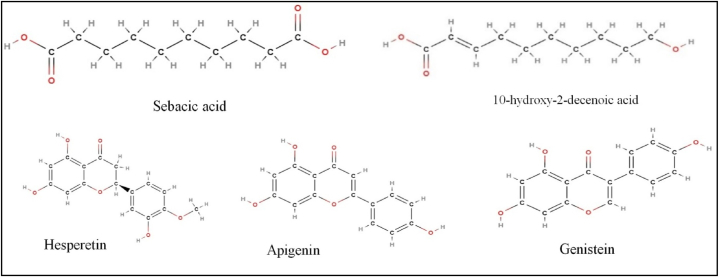
Showing the 2D structures of important lipids and flavonoids present in the royal jelly.

Lipids constitute about 7–18 % of RJ, significantly contributing to its biological activities. Predominantly composed of short hydroxy fatty acids with 8–12 carbon atoms and dicarboxylic groups, key components include 10-hydroxy-2-decenoic acid (10H2DA), and sebacic acid (SA) [17] (Fig. 4).

10H2DA plays a crucial role in caste differentiation by regulating epigenesis through the inhibition of histone deacetylases, enzymes that break down ε-acetyl-lysine residues of histones [16,18]. It has bactericidal effects against harmful bacteria like *Paenibacillus* larvae, protecting bee hives [19], and shows antibacterial effects against lipoteichoic acid from *Staphylococcus aureus* and other pathogenic bacteria linked to human colon cancer [20,21].

10H2DA also exhibits neurogenic activities by stimulating progenitor cell differentiation, mimicking neurotrophic factors [22]. It is used in cosmetics and anti-cancer drugs for its skin-whitening properties and anti-proliferative effects by suppressing transcription factors and proteins such as tyrosinase-related protein 1 (TRP-1) and TRP-2 [23]. Additionally, 10H2DA inhibits the activity of matrix metalloproteinases (MMPs), preventing tissue aging and diseases like rheumatoid arthritis [24,25].

SA and 10H2DA have anti-inflammatory properties by regulating proteins in the kappa-B signalling and mitogen-activated protein kinase (MAPK) pathways [21,26]. These acids also increase the activity of estrogen receptors ERα and ERβ, benefiting the skeletal and muscular systems [27,28].

### 5.3 Proteins

Proteins make up about 50 % of RJ, with approximately 80 % consisting of nine Major RJ Proteins (MRJPs), which have molecular weights between 49 and 87 kDa. These proteins are nutritionally valuable and play a crucial role in the development of young female larvae through cell proliferation [29]. Among MRJPs, MRJP 1 is significant; existing in heat-resistant oligomer and less resistant monomer forms [30]. The glycoprotein royalactin mimics epidermal growth factor (EGF) effects in rat hepatocytes and regulates developmental processes in bee larvae [31].

Other RJ proteins include jelleines, royalisin, and aspimin, with royalisin and jelleines being antimicrobial peptides that enhance immune responses in bee larvae. Royalisin, rich in cysteine residues, remains stable at extreme temperatures and low pH, while its antimicrobial properties are due to hydrophobic residues that disrupt bacterial membranes. Apolipophorin-3 and glucose oxidase contribute to RJ's antimicrobial properties by forming lipid-protein complexes and catalyzing glucose oxidation into hydrogen peroxide, respectively [7].

### 5.4 Phenols, flavonoids, and free amino acids

The antioxidant properties of RJ are due to polyphenolic compounds and flavonoids [32]. Key phenolic compounds include pinobanksin, dodecanoic acid, octanoic acid, and 1,2-benzenedicarboxylic acid. Flavonoids of RJ are categorized into flavanones (hesperetin, naringenin, isoakuranetin), flavonols (kaempferol, isorhamnetin), flavones (apigenin, glucoside of luteolin, chrysin, acacetin), and isoflavonoids (formononetin, genistein, coumestrol) (Fig. 4). These compounds provide anti-inflammatory and antiapoptotic properties [17]. RJ from younger larvae contains higher levels of proteins and phenolic compounds, enhancing free radical scavenging activity [32].

RJ also contains trace amino acids like valine, glutamic acid, serine, glycine, cysteine, alanine, tyrosine, phenylalanine, leucine, isoleucine, and threonine, contributing to its nutritional and biological activity [17,33]. In addition to this, RJ includes vitamins B complex, C, A, and E, with a high concentration of vitamin B5 (Pantothenic acid), linked to lifespan extension. It also contains elements like P, S, W, V, Ni, Na, Mg, Ca, Cu, Zn, Fe, Al, Sr, Pb, Hg, Ba, Bi, Cd, Sn, Cr, Mn, and Mo, with mineral salts making up about 1.5 % of its composition [34]. RJ is rich in acetylcholine, crucial for cognitive function and memory, regulated by choline acetyltransferase activity influenced by glucose and insulin metabolism, potentially preventing cognitive dysfunction [35,36]. RJ also contains nucleotides, free bases, phosphates, ADP, ATP, and AMP, which are essential for energy production and enzymatic actions [37]. AMP N1-oxide, unique to RJ, promotes neurite outgrowth and PC12 cell differentiation into neurons, mimicking nerve growth factor activity through MAPK/ERK1/2 and PI3K/Akt pathways [17,38].

## 6 Pharmacological importance of RJ

**Fig. 5:**
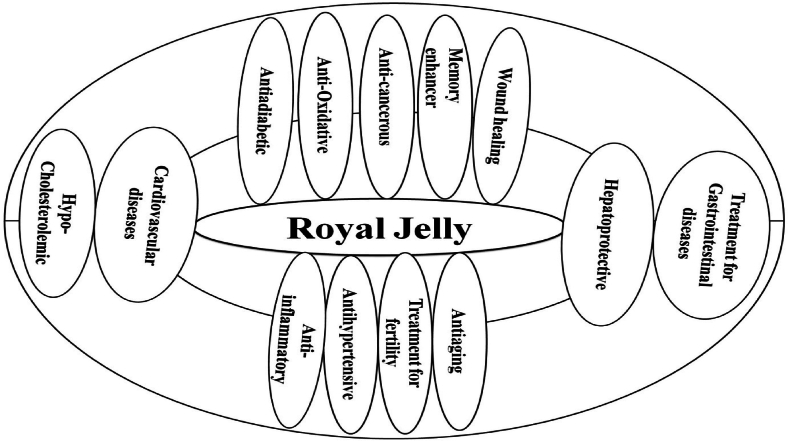
Depicts the different pharmacological and biologically defensive properties of royal jelly.

RJ exhibits numerous properties which are known to have beneficial effects on humans like cardiovascular disease [39], antihypertensive activity [40], hypo-cholesterolemic activity [41], anti-aging [42], anti-cancerous [43], memory enhancer [44], hepatoprotective [45], anti-obesity [46], anti-diabetic [47], wound healing [48], anti-inflammatory and antioxidative [49]. Each constituent of RJ has shown its advantages regarding human welfare and acted as an excellent therapeutic agent against various diseases (Fig. 5 and Table 6).

**Table 6:** This table summarizes the diseases, their causes and symptoms, the properties of royal jelly relevant to each condition, effective bioactive compounds found in royal jelly, and the biological properties of royal jelly relevant to each condition.

Disease	Causes	Symptoms	Property of Royal Jelly	Effective Bioactive Compound	Biological Property
Ulcerative Colitis	Chronic, inflammatory bowel disease	Abdominal pain, diarrhea, rectal bleeding	Inhibition of pro-inflammatory cytokines TNF-α and IL-1β, elevation of anti-inflammatory cytokine IL-10	Royalactin, Royalisin	Anti-inflammatory, boosts activity of IL-10, decreases T-cell proliferation, inhibits TNF-α and IL-1β
Crohn's Disease	Chronic, inflammatory bowel disease	Abdominal pain, diarrhea, weight loss	Inhibition of pro-inflammatory cytokines TNF-α and IL-1β, elevation of anti-inflammatory cytokine IL-10	Royalactin, Royalisin	Anti-inflammatory, boosts activity of IL-10, decreases T-cell proliferation, inhibits TNF-α and IL-1β
Lactose Intolerance	Lack of β-galactosidase, maldigestion of lactose from milk and milk products	Abdominal pain, diarrhea, flatulence	Synergism with probiotic yogurt, boosting probiotics in fermented milk products	Lactobacillus helveticus	Enhances probiotic activity, aids in lactose digestion
Chronic Diarrhea	Various causes including infections, dietary issues, and intestinal disorders	Frequent, loose stools, abdominal pain	Anti-diarrheal potency due to antimicrobial activity of royalisin and royalactin	Royalisin, Royalactin	Antimicrobial, anti-diarrheal
Chronic Constipation	Lack of fiber, dehydration, sedentary lifestyle, medications	Straining during bowel movements, infrequent bowel movements	Acetylcholine in RJ induces smooth muscle contractions, antimicrobial activity of royalisin and royalactin	Acetylcholine, Royalisin, Royalactin	Induces smooth muscle contractions, antimicrobial, potentially aids in bowel movements
Gastric Ulcers	NSAID use, H. pylori infection, excessive alcohol consumption	Abdominal pain, bloating, heartburn, nausea	Normalization of gastric tissues via increase of PGE-2 and COX-2, reduction of MPO and iNOS	Not specified	Normalizes gastric tissues, reduces inflammation
Intestinal Ulcers	NSAID use, H. pylori infection, excessive alcohol consumption	Abdominal pain, bloating, heartburn, nausea	Attenuation of pro-inflammatory cytokines TNF-α and IL-1β, reduction of lipid peroxidation, augmentation of endogenous antioxidant enzymes SOD and CAT	Not specified	Reduces inflammation, increases antioxidant activity
Peptic Ulcers	NSAID use, H. pylori infection, excessive alcohol consumption	Abdominal pain, bloating, heartburn, nausea	Reduction of gastric ulcers via anti-inflammatory action, attenuation of pro-inflammatory cytokines TNF-α and IL-1β, augmentation of antioxidant enzyme activity	Not specified	Reduces inflammation, increases antioxidant activity
Nicotine-Induced Gastric Mucosal Changes	Ingestion of nicotine, leading to mucosal damage	Abdominal discomfort, changes in mucosal integrity	Amelioration of mucosal changes via unknown mechanisms	Not specified	Unknown, possibly anti-inflammatory

### 6.1 Neuroprotective action of RJ

**Fig. 6:**
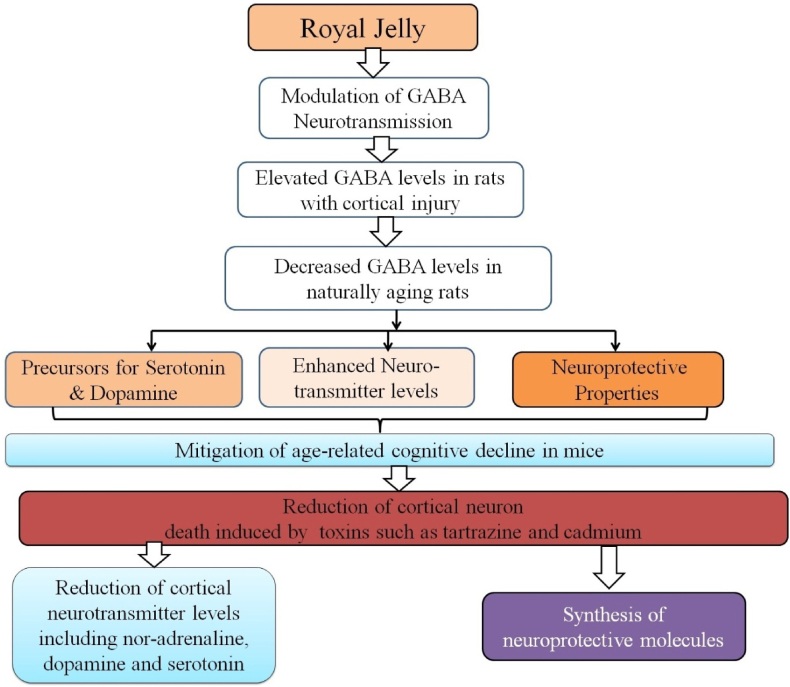
Showing the neuroprotective effect of propolis, royal jelly modulates the neurotransmission by stimulating the gamma amino butyric acid (GABA) in the brain which in turn stimulates the synthesis of serotonin and dopamine, increases the levels neurotransmitters, and hence acts as neuroprotective natural agent. In addition to this administration of royal jelly increases the synthesis and secretion of other neurotransmitters and also some neuroprotective molecules.

RJ has demonstrated numerous beneficial effects on the nervous system, including memory enhancement, increased energy levels, prevention of senility, anxiety reduction, and calming effects on hyperactivity. Its neuropharmacological actions involve modulating gamma-aminobutyric acid (GABA) neurotransmission, a crucial regulator of brain activity. RJ influences GABA-transaminase (GABA-T), the enzyme metabolizing GABA, which is synthesized from glutamate by glutamate decarboxylase (GAD) [50]. Studies indicate RJ administration elevates GABA levels in rats with tartrazine-induced cortical injury, suggesting neuroprotective effects, but decreases GABA levels in the striatum and hypothalamus of aging rats, showing complex regulation of GABAergic neurotransmission (Fig. 6).

RJ also contains tryptophan and tyrosine, precursors for serotonin and dopamine, crucial for mood and cognitive function [51,52]. Treatment with RJ and tyrosine in experimental models increased brain dopamine levels and its metabolites [53,54], enhancing cognition through improved neurotransmission. RJ mitigates age-related cognitive decline induced by d-galactose in mice, restoring brain noradrenaline and dopamine levels [55], indicating its neuroprotective potential (Fig. 6).

RJ reduces cortical neuron death from toxins like tartrazine and cadmium and restores levels of neurotransmitters such as noradrenaline, dopamine, and serotonin, supporting neuronal integrity. It also stimulates neuroprotective molecule synthesis, evidenced by increased cysteic acid levels in aged rats [56,57], suggesting involvement in the cysteine-taurine metabolic pathway [58]. The beneficial effects of RJ on the nervous system likely stem from neurotransmission modulation, neuroprotection against age-related cognitive decline, and stimulation of neuroprotective pathways, underscoring its potential as a supplement and therapeutic agent for cognitive enhancement and neuroprotection (Fig. 6).

### 6.2 RJ as an anti-aging remedy

**Fig. 7:**
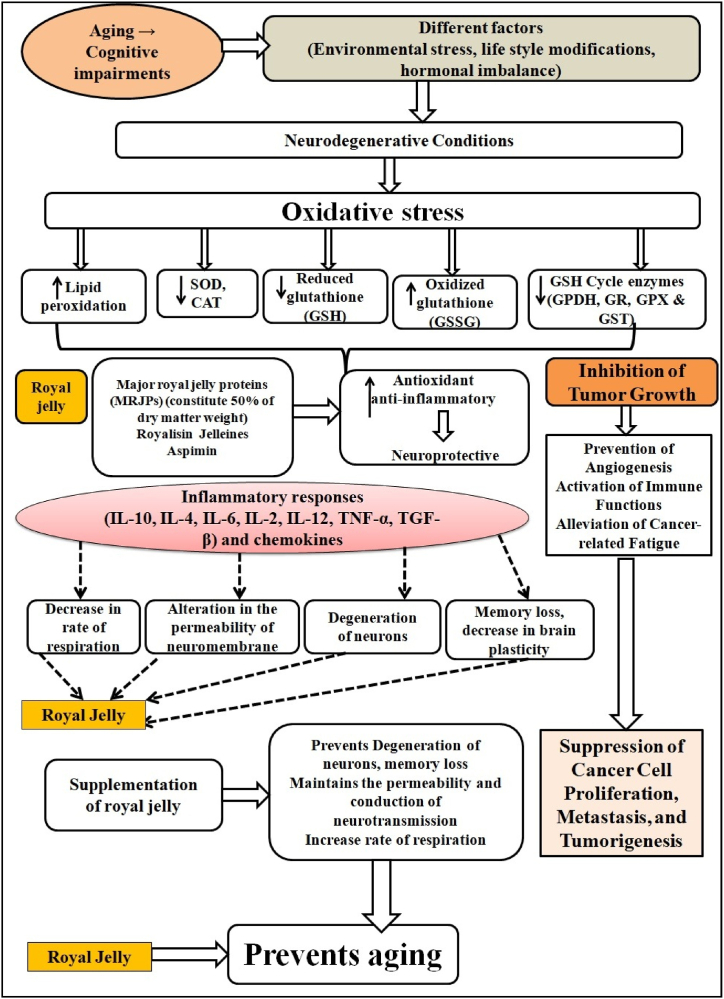
Royal jelly (RJ) is used as anti-aging by preventing the neurodegeneration through the reduction in oxidative stress, because RJ increases the GSH content and accelerating the activities of antioxidative enzymes such as SOD, CAT, G6PDH, GR, GPX and GST. Moreover, RJ decreases the secretion of inflammatory cytokines by down-regulating the inflammatory pathways and hence protects the neurons from damages and prevents the aging. Royal jelly suppresses the progression and development of tumors by inhibiting te angiogenesis, activation of immune system and prevents the metastasis and tumorgenesis.

According to Halloran et al. (2012), aging is a major risk factor for neurodegenerative diseases [59], notably Alzheimer's disease (AD), which is characterized by progressive loss of working and situational memory [60]. Previous studies reported that RJ can protect spatial memory in rats modeled with sporadic AD through an intracerebroventricular injection of streptozotocin (icv-STZ) by enhancing hippocampal neurogenesis and reducing oxidative stress and neurodegeneration [36,61] (Fig. 7).

In AD models, such as 10-month-old APP/PS1 mice, RJ improves memory by preventing neuronal death and modulating the cAMP/PKA/CREB/BDNF pathway [62]. Furthermore, RJ ameliorates memory deficiencies in ovariectomized (OVX) rats and improves memory in OVX cholesterol-fed rabbits by regulating oxidative stress and cholinergic neurotransmitter levels [63,64].

RJ also affects neurotransmitter levels in the prefrontal cortex, influencing cognitive processes related to working memory (WM) [65]. Long-term RJ administration reduces striatal GABAergic transmission and GABA concentration in aged Wistar male rats, enhancing dopamine transmission activity and spatial memory [56]. MRJPs specifically enhance spatial memory by modulating cysteine, taurine, and energy metabolism [66]. Thus, RJ shows significant potential in improving memory function through multiple mechanisms.

### 6.3 Effect of RJ on fertility

Male infertility is influenced by factors such as smoking, alcohol consumption, sexual behavior, and diet. Primary infertility prevalence among couples ranges from 13.2 % to 17.3 %, with ovulatory problems accounting for 39.7 % and male factors 29.1 % [67]. Male factors alone are responsible for at least half of all infertility cases [68], with estimates suggesting a prevalence of 40.9 % [69]. Male factors contribute to infertility in 20–70 % of cases, with infertile men comprising 2.5 %–12 % of the population [70].

RJ has shown benefits for human fertility, including improvements in hormone balance, sperm, and ovule quality [71]. [72] RJ supplementation elevates testosterone levels, ejaculate volume, seminal fructose, sperm motility, and sperm count in male animals [10]. Cryopreservation of sperm reduces viability, but RJ treatment at specific concentrations can enhance sperm viability over time.

RJ significantly improves total sperm motility in chilled and frozen-thawed ram sperm due to its high calcium ion concentration. RJ administration at doses of 50, 100, and 150 mg/kg increases sperm motility in animal models like rabbit bucks and mice [73]. Thus, RJ is a promising natural supplement for enhancing male fertility, but optimal dosage and administration methods need further research to maximize its efficacy.

### 6.4 Anti-tumor action

RJ, a bee secretion, contains compounds like 10-hydroxy-2-decanoic acid (10H2DA) and proteins such as apalbumin-1 and apalbumin-2, which inhibit cancer progression (Fig. 7). 10H2DA can inhibit VEGF, reducing angiogenesis, cell proliferation, and migration, thus hindering tumor vascularisation [74]. RJ also has antioxidative properties, enhancing the production of antioxidants like GSH, GSH-Px, SOD, and GST in the kidneys and liver of rats [75] (Fig. 7). This antioxidative effect is synergistic with Cisplatin, an anti-cancer agent, helping combat oxidative stress-induced cancer. RJ inhibits tumor growth, prevents angiogenesis, activates immune functions, and alleviates cancer-related fatigue when supplemented in cancer patients. It shows promise as an adjunctive treatment during menopause for breast cancer by suppressing cancer cell proliferation, metastasis, and tumorigenesis through inhibition of angiogenesis and immune system stimulation [76,77].

### 6.5 Antibiotic effect

Royalisin, a protein in RJ, exhibits strong antibacterial properties against various Gram-positive bacteria but not Gram-negative bacteria [78]. Direct ingestion of RJ can degrade its active components due to pH changes [19,49]. Jelleines, also in RJ, show antimicrobial actions: Types I, II, and III act against Gram-positive bacteria, Gram-negative bacteria, and yeast, respectively, while Type IV does not [79]. Some MRJPs (2–5) have antibacterial activity against *E. coli*, a Gram-negative bacterium.

Bilikova et al. (2015) found that royalisin and royalisin-D significantly reduced survival rates of *S. intermedius* and *P. aeruginosa*, Gram-negative and Gram-positive bacteria, respectively, with royalisin showing higher efficacy [80]. Conversely, Hasan et al. (2020) found RJ had antimicrobial activity against *E. coli*, unlike gentamicin and its combination with doxycycline, which were ineffective [81]. MRJPs (2–5) and 7 produced recombinantly demonstrated consistent antibacterial activity [82]. RJ also showed antibacterial activity against *S. aureus* when infused into milk, enhancing the properties of milk [83]. Furthermore, RJ was more effective against anaerobic periodontopathic bacteria than aerobic ones in a study comparing RJ and chlorhexidine [84]. Hence, RJ and its components exhibit significant antimicrobial properties, particularly against Gram-positive bacteria and certain Gram-negative strains, with varying efficacy depending on the specific proteins and conditions used.

### 6.6 Wound healing effect and anti-inflammatory action

**Fig. 8:**
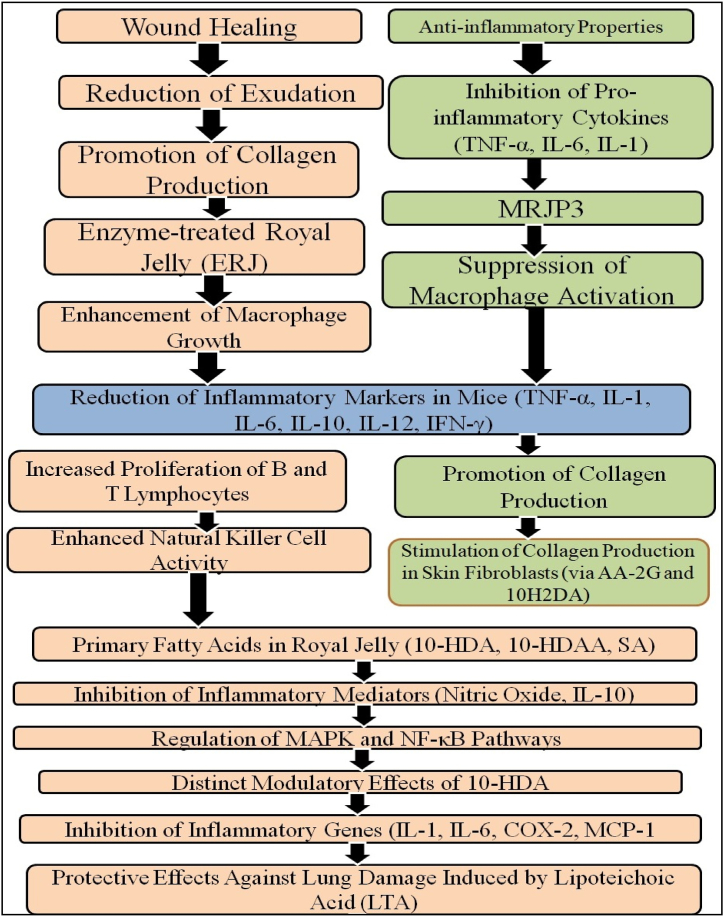
Royal jelly (RJ) inhibits the inflammatory pathways by declining the synthesis and secretion of pro-inflammatory cytokines (TNF-α, IL-6 and IL-1) by suppressing the activation of macrophages. In addition to this RJ regulates the inflammatory secretion and promotes the production of collagen. Royal jelly (RJ) helps in the healing of wounds by promoting the production of collagen because royal jelly contains various enzymes which accelerate the healing of wounds. Moreover, reduction in the secretion of proinflammatory cytokines (TNF-α, IL-6 and IL-1) by suppresses the activation of macrophages.

The cosmetic industry often incorporates RJ due to its antioxidative, antibacterial, anti-inflammatory, and wound-healing properties. RJ has been shown to prevent the release of pro-inflammatory cytokines like TNF-α, IL-6, and IL-1 in mouse macrophage cultures without harming the cells (Fig. 8) [85]. Components of RJ, such as MRJP3, regulate cytokine release. It also contains ascorbic acid-2-O-α-glucoside (AA-2G), which stimulates collagen production, and 10H2DA, which enhances TGF-β1 release, further promoting collagen production and wound healing [86] (Fig. 8).

A study on enzyme-treated RJ (ERJ) showed it supports macrophage growth and protects against lipopolysaccharide (10.13039/501100012274LPS)-induced stress. ERJ reduced inflammatory markers (TNF-α, IL-1, IL-6, IL-10, IL-12, IFN-γ) and boosted B and T lymphocyte proliferation and natural killer cell activity in mice (Fig. 8). Previous studies have investigated RJ's fatty acids (10-HDA, 10-HDAA, Sebacic acid) and found they inhibit key inflammatory mediators like nitric oxide and IL-10 in a dose-dependent manner, with Sebacic acid also reducing tumor necrosis factor alpha (TNF-α) secretion [20]. These fatty acids modulate inflammatory genes and pathways, including MAPK and NF-κB. It has been reported that 10-HDA's anti-inflammatory effects by reducing the expression of genes such as IL-1, IL-6, COX-2, and MCP-1 in vitro. In mice, a 100 mg/kg dose of 10-HDA reduced lung damage and inflammatory cytokines (IL-10, MCP-1, TNF-α) caused by lipoteichoic acid (LTA) [62]. Therefore, RJ and its components show significant potential for treating inflammatory conditions and enhancing skin health (Fig. 8).

### 6.7 Effect on gastrointestinal diseases

Gastrointestinal disorders, including irritable bowel syndrome, peptic ulcers, liver diseases, pancreatitis, gallstones, and Crohn's disease, are prevalent, especially in tropical regions [87]. It has been studied that functional gastrointestinal issues affect about 40 % of people globally [88]. Dietary patterns significantly influence these conditions, with a Westernized diet high in carbohydrates and animal proteins linked to chronic inflammatory bowel diseases [89]. The transition from urban to rural settings impacts gut microbiota composition [90].

The gastrointestinal tract's role in nutrient absorption and immune response increases the risk of inflammatory, autoimmune, and chronic conditions [91]. RJ is noted for its therapeutic potential against inflammation, liver disease, hypercholesterolemia, oxidative stress, and immune diseases [92]. RJ contains bioactive compounds, including proteins, vitamins, phenolics, and flavonoids, with its major protein constituents (MRJPs) highlighted as significant therapeutic agents [93,94].

### 6.8 Immunomodulatory effects

RJ exhibits diverse pharmacological activities, particularly immunomodulatory effects. Oka et al. (2001) found that RJ inhibits mast cells in DNP-KLH mice, reducing antigen-specific IgE and histamine production while restoring macrophage function and enhancing Th1/Th2 response [95]. In β-lactoglobulin allergic mice, RJ reduces serum anti-β-Lg IgE, IgG, and histamine, mitigating allergic symptoms [96].

RJ also suppresses atopic dermatitis-like lesions in NC/Nga mice by modulating IFN-γ production and iNOS expression [97]. In SLE-prone mice, RJ lowers serum IL-10, anti-ssDNA, dsDNA, erythrocyte autoantibody levels, and splenic autoreactive B cells [98]. In children with SLE, RJ increases CD4^+^ and CD8^+^ regulatory T cells while decreasing CD4^+^ T cells, reducing disease severity [99].

In Graves' disease, RJ decreases TNF-α in lymphocytes and increases IFN-γ and Th1/Th2 cytokine ratios [100]. Protease-treated RJ enhances mucosal IgA responses by promoting antigen uptake by M cells [101]. MRJP3 and apolipoprotein III-like protein in RJ exhibit immunoregulatory activity; MRJP3 suppresses T-cell IL-4, IL-2, IFN-γ production and reduces anti-OVA IgE and IgG1 levels [102]. Apolipoprotein III-like protein enhances immune response post-phosphorylation [103].

**Fig. 9:**
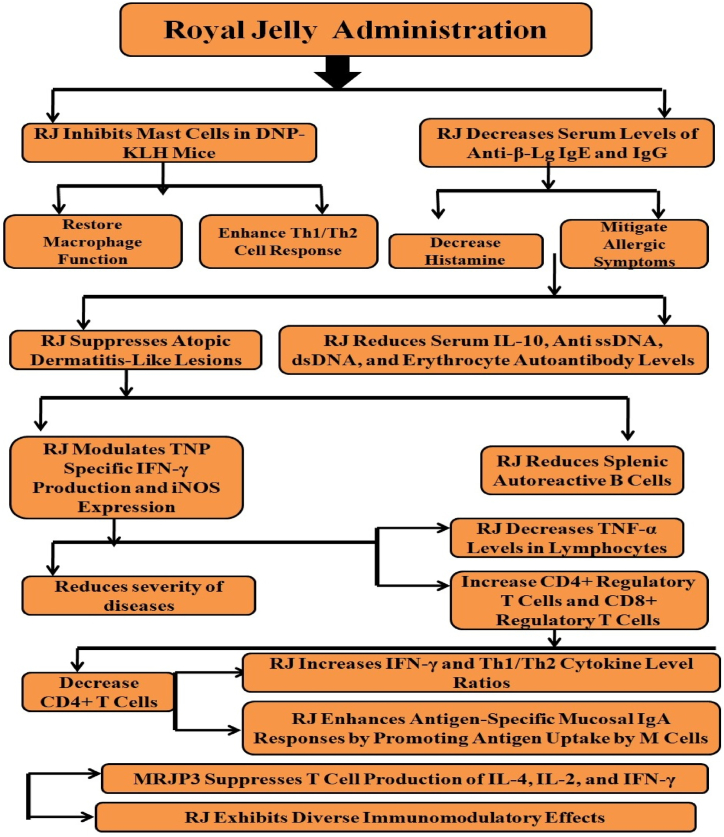
Royal jelly (RJ) shows immunomodulatory effect against the various immune compromising diseases by decreasing the secretion and synthesis of different immune biomolecules. Moreover, major royal jelly proteins (MRJPs) suppresses the production of IL-4, IL-2 and IFN-γ thereby drives the immunomodulatory effects.

10-HDA, a component of RJ, shows mixed immune effects: it reduces IL-6 production and NF-κB activation but inhibits T cell proliferation and antigen-specific responses [[104], [105], [106]]. However, it can restore body, thymus, and spleen weights in cyclophosphamide-induced mice, improving the thymus/spleen index and enhancing T and B cell activity [107] (Fig. 9).

### 6.9 Reduce liver damage

RJ demonstrates significant hepatoprotective properties. It regulates 267 liver genes in mice, decreasing squalene epoxidase (SQLE) and increasing low-density lipoprotein receptor (LDLR), thus reducing cholesterol levels [108]. RJ also upregulates genes encoding GST and GSH-Px [108].

Liver function, crucial for metabolism, is often impaired by toxic chemicals and drugs, marked by elevated alanine aminotransferase (ALT) and aspartate aminotransferase (AST) levels [109]. Toxicants like cadmium and CCl_4_ lower glutathione (GSH) and increase malondialdehyde (MDA). RJ restores antioxidants (SOD, CAT and GSH) and reduces AST and ALT, mitigating liver damage [110,111]. RJ also alleviates liver injury from azathioprine and paracetamol [112,113].

RJ counteracts cisplatin (CDDP) and taxol (TXL)-induced liver injury by restoring GST and GSH-Px levels and reducing hepatocyte apoptosis [114]. It lowers ALP and LDH levels elevated by TXL and modulates liver growth regulatory factors [115]. RJ diminishes iNOS and IL-1β expression while upregulating Nrf2 and Bcl-2, and down regulating caspases-3 and Bax, protecting against cadmium toxicity [116]. RJ components like MRJP2 protect against CCl_4_-induced liver injury by mitigating the oxidative in liver by enhancing antioxidant capacity [117]. Thus, RJ safeguards the liver from various injuries by enhancing its antioxidant defenses.

### 6.10 Diabetes

Diabetes, particularly Type 2 Diabetes (T2DM), results from insulin secretion and resistance issues, affecting adipose tissue, liver, and skeletal muscle [118,119]. T2DM is linked to liver diseases like hepatic cirrhosis, hepatocellular carcinoma, and nonalcoholic fatty liver disease, emphasizing the liver's role in glucose and lipid homeostasis [120,121].

RJ has demonstrated beneficial effects in managing diabetes. In women with T2DM, RJ reduces serum fasting blood glucose and glycosylated hemoglobin levels while increasing insulin concentration, thereby reducing the risk of complications [122,123]. RJ also raises serum apolipoprotein A-I (ApoA-I) levels and improves the ApoB/ApoA-I ratio in T2DM patients (Khoshpey et al., 2016).

In STZ-induced diabetic rats, RJ lowers fasting blood glucose, AST, ALT, and ALP levels, while increasing insulin, albumin, and total protein levels [124]. Additionally, RJ enhances total antioxidant capacity and reduces insulin resistance in diabetic patients [125]. Long-term RJ administration inhibits glucose-6-phosphatase, improving hyperglycemia by increasing adiponectin and adiponectin receptor 1 mRNA expression, and activating AMP-activated protein kinase in abdominal fat [119].

### 6.11 Restrain obesity

Obesity, a chronic condition influenced by various factors, is marked by heightened oxidative stress and prolonged activation of macrophages in peripheral tissues. Addressing inflammation and oxidative stress is key to effective treatment. RJ supplementation in overweight adults has shown promising results, reducing total cholesterol, C-reactive protein, and increasing adiponectin, serum total antioxidant capacity, bilirubin, and leptin, thus benefiting overweight individuals [126]. Studies in mice fed a high-fat diet and supplemented with RJ observed reduced inflammation and elevated levels of Irisin, promoting metabolic thermogenesis in brown adipose tissue [127,128]. Despite 10-HDA being a significant component of RJ, it was found ineffective in preventing obesity [129].

### 6.12 Anticancer

RJ shows promising potential in cancer treatment. RJ improves myelosuppression in mice with Ehrlich ascites tumors by inhibiting spleen hematopoietic function and enhancing survival rates, likely through reducing prostaglandin E2 [130]. Prostaglandin E2 regulates lymphocyte proliferation, inhibits macrophage tumoricidal activity, and modulates immune responses [130]. RJ inhibits the proliferation of human breast cancer MCF-7 cells induced by bisphenol A [131] and reduces glutathione levels while increasing lipid peroxidation in human Caco-2 cells when combined with interferon α [132]. In a 4T1 breast cancer mouse model, RJ treatment reduces tumor weight and enhances antioxidant activity in the liver, kidney, and serum [133]. RJ extracts also show strong cytotoxicity against human glioblastoma multiforme [134] and HeLa cells [135].

Lipophilic extract of RJ inhibits human neuroblastoma cell proliferation [136]. Components such as AMP-N1 oxide promote axonal growth and activate the MAPK signaling pathway [23]. MRJP2 promotes caspase-dependent apoptosis in HepG2 cells [137], while 10-HDA inhibits melanin production in B16F1 melanoma cells [23]. The derivative HPO-DAEE induces apoptosis in human lung cancer cells via the ROS-ERK-p38 and CHOP pathways [138].

## 7 Importance of RJ in health of animals

Numerous studies have investigated the effects of RJ on animal health. RJ enhances cartilage development due to its high collagen content and strengthens bone and tooth structures due to calcium and selenium. It protects blood cells, heart, liver tissues, muscles, and the nervous system due to its potassium content. RJ improves wound healing; a study on mice showed daily RJ application significantly increased wound healing compared to controls, suggesting RJ showed significantly higher effect as compared to Nitrofurazone [139]. RJ also enhanced the healing of tympanic membrane perforations in guinea pigs [140]. In reproductive health, RJ increased sperm motility, reduced abnormal sperm rates, and improved sperm quality in animals [141]. It inhibited age-related testosterone decline in old hamsters [142] and mitigated the negative effects of stress on male rabbit fertility during hot conditions, improving various sperm parameters and plasma biochemical markers [143]. RJ has nephroprotective effects, preventing cisplatin-induced kidney damage [144]. In diabetic rats, RJ alleviated oxidative stress and improved liver and pancreas biochemical markers [145]. It also protects against cadmium-induced genotoxicity and oxidative stress in rats [146]. RJ mitigated the adverse effects of aluminum chloride on reproductive and hormonal health in poisoned rats [147]. It improved sperm parameters and reduced oxidative stress in male rats exposed to hydrogen peroxide [148]. RJ increased ovulation rates and estrus onset in animals [147,149].

Supplementation with bee products improved immunity and performance in quail [150,151]. RJ reduced hyperglycemia and body weight in obese/diabetic mice [119], prevented hyperlipidemia, and improved blood clotting levels [152]. It also reduced epidural fibrosis after laminectomy in rats [153] and delayed atheroma formation in hyperlipidemic rabbits. RJ exhibited osteo inductive and anti-inflammatory effects in treating periodontal diseases [154] and reduced osteoporosis-related bone loss in oophorectomized rats [155]. It restored immune function and improved premature mortality in mice with low micronutrient intake [156]. RJ enhanced spatial memory and inhibited cognitive impairment in older rats [65,66].

RJ showed immunomodulatory effects in the 4T1 breast cancer model in mice, enhancing TNF-a and IgG levels and improving kidney cell size [133]. It reduced breast tumor development and enhanced antioxidant capacity [157]. RJ also reduced the size of WEHI-164 fibrosarcoma tumors in mice [158]. RJ protected cardiac muscle from ischemia, enhancing contraction activity and coronary blood flow. It alleviated physical fatigue in mice [159] and modulated disorders in a Parkinson's disease model in rats [160] RJ protected the colonic mucosa from acetic acid-induced damage in rats, reducing mast cell numbers and colonic erosion [161]. It decreased oral mucositis in rats subjected to radiotherapy [162]. RJ reduced corticosterone levels and improved the antioxidant defense system in stressed rats [163]. RJ, propolis, and bee pollen showed significant antibacterial effects against *Aeromonas hydrophila* and *Vibrio cholerae*, suggesting potential in controlling pathogenic bacteria [164].

### 7.1 RJ: A natural boost health for human

RJ is known for its benefits in cell regeneration, metabolism, and vitality. Rich in natural hormones, vitamins, essential fatty acids, amino acids, sterols, phosphorous compounds, and acetylcholine, RJ aids nerve message transmission and endocrine function. Its nucleic acids and collagen components provide anti-aging effects (Table 7). Gammaglobulin in RJ strengthens the immune system, while 10-HDA has strong antibiotic properties [165,[166], [167], [168], [169], [170]].

**Table 7:** Dosage of royal jelly for human use.

Condition	Dose	Effect of Royal Jelly	References
Elderly Physical Performance and Memory	Not specified	Slows muscle strength deterioration and helps preserve memory	[65,171]
Skin Protection	Not specified	Increases collagen production, protects against UVB-induced photoaging	[165]
Infection and Inflammation	Not specified	Inhibits Pseudomonas aeruginosa adhesion, protects from inflammatory responses, and offers protection against Fumonisin toxicity	[113,172]
MRSA Infections	Not specified	Potential alternative treatment	[173]
Dry Eye Syndrome	Not specified	Increases tear secretion, preserves lacrimal gland function	[174,175]
Upper Respiratory Tract Infections	Not specified	Suggested as supplements for treatment	[176]
Cardiovascular Health	350 mg per capsule	Reduces serum total cholesterol and LDL levels	[177]
	6 g per day for 4 weeks	Lowers small VLDL levels	[178]
Glucose Metabolism	20 g	Improves glucose tolerance	[179]
Autoimmune Diseases	Not specified	Improves clinical severity scores and laboratory markers in systemic lupus erythematosus	[99]
Dental Health	0.2 % RJ	Antibacterial effects	[180,181]
Allergies	Not specified	Reduces intestinal anaphylactic responses and histological lesions caused by β-Lg sensitivity	[96]
Thyroid Health	Not specified	May have anti-thyroid effects, beneficial for Graves' disease	[85]
Skin Pigmentation	Not specified	Inhibits melanogenesis, suggesting use in skincare products	[23]
Liver Health	Not specified	Protects against alcohol-induced hepatomegaly, restores transaminase levels	[182]
	Not specified	Offers hepatoprotection against paclitaxel-induced toxicity	[115]
Skin Hydration	520 mg per day for 10 weeks	Improves epidermal hydration and ceramide levels	[183]
Diabetic Foot Ulcers	Not specified	Aids in healing diabetic foot ulcers	[184]
Kidney Health	Not specified	Antioxidants positively impact renal damage caused by oxidative stress and inflammation	[185]
Athletic Performance	Not specified	Improves body height, muscle mass, reduces fat components	[186]
Pain Relief	200 mg/kg	Analgesic effects comparable to aspirin for acute pain, more effective than aspirin for chronic pain	[187]
Breast Cancer	Not specified	Inhibits growth-promoting effect of Bisphenol A on MCF-7 breast cancer cells	[131]

RJ mitigates damage from 5-fluorouracil and exhibits antitumor, antibiotic, immunomodulatory, estrogenic, and neurogenic activities [188,189]. A six-month intake of RJ improves erythropoiesis, glucose tolerance, and mental health in humans [190]. It significantly improves sperm count and motility, aiding infertility treatment [170] and is effective in treating early menopause and ovarian damage caused by Adriamycin [191]. For chronic conditions like menopausal osteoporosis and cardiovascular disorders, 150 mg of RJ for three months improves lipid profiles and may control menopause-related dyslipidemia [192]. RJ also improves the quality of life for postmenopausal women, treating sexual and urinary dysfunctions [193]. A 1000 mg capsule of RJ for two months can reduce pre-menstrual syndrome (PMS) [194].

RJ, combined with Indian honey, positively affects fetal membranes in cases of early rupture [195]. RJ contains major fatty acids like 10-H2DA, 10-HDAA, and SEA, which have anti-inflammatory effects and modulate key inflammatory pathways [26]. RJ stimulates the central nervous system, enhancing muscle tone and activity, and promotes neurotrophic effects via Glial Cell-Derived Neurotrophic Factor (GDNF) production, aiding brain cell differentiation and function [196]. RJ improves serum antioxidant capacity and insulin resistance in diabetes [122,125,197] and significantly impacts glycemic control in Type 2 diabetics [198]. It may aid in weight management in diabetic women [199]. RJ supports fatigue recovery in cancer patients [199] and improves oral mucosa symptoms during radiotherapy and chemotherapy, shortening healing times [200]. RJ may protect against radiation-induced apoptosis in human blood leukocytes [201]. RJ and honey exhibit antioxidant effects, reducing Cisplatin-induced nephrotoxicity in cancer patients [202].

#### 7.1.1 Dosage of RJ for human use

Preparation and formulation of dosage of RJ varies from individual to individual as well as from one age group to another age group. In table, 8 dosage of RJ has been summarized (Table 8).

**Table 8:** Dosage of royal jelly for human use.

Group	Condition/Need	Dosage	Supplement Constituents	Reference (s)
Infants	Growth and development, strengthen immunity and nervous system	0.5 g/day for 2–12 months	Raw royal jelly	[[203], [204], [205], [206], [207]]
Premature Infants	Complications of prematurity	50 mg to 1 g/day		
	Complications of prematurity	Medium dose of 0.25 g/day		
Children (1–5 yrs)	Low immune system, nervous system impairment, weakness, loss of appetite, anorexia, anaemia	0.5 g/day	Royal jelly	
Children (5–12 yrs)	Low immune system, nervous system impairment, weakness, loss of appetite, anorexia, anaemia	0.5–1 g/day		
Children (1–5 yrs)	Acute infection and colds	2.5 g/day for 1–3 days		
Children (5–12 yrs)	Acute infection and colds	5 g/day for 1–3 days		
Adults	Immunity, insomnia, skin disorders, anaemia, low libido, hormonal imbalance, wounds, premenstrual syndrome, menopause, osteoporosis	1–2 g/day		
	Diabetes, depression, Hashimoto's disease, arthritis	3–5 g/day		
	Recent depression	Up to 10 g/day for 10 days/month for 3 months		
	Immunity, convalescence, preparation for surgery, autoimmune diseases, cancer, hormonal imbalances, infertility, ovarian cyst, uterine fibroma, thyroid problems	1 g/day, up to 10 g/day for 1–3 days in beginning of colds or acute infections, up to 3–5 days post-op healing	Royal jelly, bee products, plants	
	Heavy working conditions	10 g/day	Royal jelly	
	Early onset of colds	10 g/day for 1–3 days		
	Post-operative healing	5–10 g/day for 3–5 days		
	Side effects of chemotherapy	3 g/day for 6–8 weeks		
	Neurodegenerative diseases, multiple sclerosis, Parkinson's disease	10–15 g/day		
Adults	Severe acne	Topical application with Boswellia essential oil; oral treatment with propolis	Royal jelly, Boswellia essential oil, propolis	[85,[208], [209], [210]]

### 7.2 Role of RJ in diet formulation

Bee products like RJ, offer essential nutrients crucial for health, especially amid rising environmental pollution and inadequate diets. RJ is rich in 10-HDA, B vitamins, and folate, provides significant nutrition (Lab Reference: CS20133271). While natural folate in RJ is vital, synthetic folic acid supplements may pose risks during pregnancy [211]. Preventive and cell-regenerative actions of RJ are supported by apinutrition (incorporation of bee products to promote overall health and vitality). These functional foods, prominent due to their ease of assimilation and pharmacological benefits are widely consumed [132,188,210,211].

RJ has demonstrated efficacy in alleviating allergic symptoms, managing cholesterol, and supporting conditions like muscular dystrophy, MS, and Parkinson's disease. It aids immune function during radiotherapy and chemotherapy, replenishing cells destroyed by treatment. With its rich amino acid and gamma globulin content, RJ strengthens the immune system against viral infections. It also contains essential nutrients supporting various health aspects, including energy, immunity, cardiovascular health, and mental well-being [180,[212], [213], [214], [215]]. Additionally, RJ acts as an adaptogen enhancing fertility and potentially extending lifespan without disease [214].

## 8 Significance

This review article provides a comprehensive critical appraisal of the pleiotropic therapeutic effects of RJ, underscoring its potential as a multifaceted natural remedy and food supplements. Following the systematic evaluation of the current scientific literature, it was elucidated that RJ has diverse biological activities anti-diabetic, anti-inflammatory, antioxidant, antimicrobial, and anti-cancer properties. It highlights the molecular mechanisms underlying these effects, thereby advancing our understanding of how RJ can contribute to health and disease management. This critical assessment and summarization of the published findings could play a significant support for both clinical and research communities to identify the gaps in existing literature, propose directions for future research, and support the development of RJ-based therapeutic strategies. The findings of this review emphasize the importance of integrating RJ into complementary and alternative medicine, potentially leading to innovative approaches in treating various ailments as well as food supplements.

## 9 Novelty

This systematic review uniquely synthesizes recent evidence on the multifaceted therapeutic potential of royal jelly, highlighting its emerging role not only as a functional food supplement but also as a promising intervention in various health conditions. Moreover, this review underscores the novel bioactive components of royal jelly and their biological activities such as anti-inflammatory, antimicrobial (antibacterial, antifungal and antiviral) and antibiotic. In addition this RJ and its metabolites having various health benefits such as gastrointestinal protection, cardiovascular, anti-tumor, anti-ageing, neuroprotective (e.g., Alzheimer's, Parkinson's), hepatotoxicity, metabolic disorders (e.g., diabetes, obesity, hyperlipidaemia), reproductive disorders (e.g., PCOS, infertility and oligospermia) and wound-healing activities. Mechanisms of action of RJ and its therapeutic efficacy offer comprehensive perspective that could guide future research and clinical applications.

## 10 Conclusion

This review critically appraises the pleiotropic therapeutic effects of RJ, demonstrating its vast potential as a natural therapeutic agent. The comprehensive analysis reveals that RJ exhibits significant anti-inflammatory, antioxidant, antimicrobial, and anti-cancer properties, supported by various molecular mechanisms. Therefore, RJ holds promise for incorporation into complementary and alternative medicine, offering a multifaceted approach to health and disease management.

## Limitations

This review, while comprehensive, faces several limitations. First, the heterogeneity of study designs and methodologies in the existing literature complicates the synthesis of findings and may introduce bias. Second, many studies on the therapeutic effects are preclinical trial on RJ, necessitating cautious extrapolation to human applications. Moreover, the variability in the composition of RJ due to differences in bee species, diet, and environmental factors poses challenges in standardizing dosages and formulations. Lastly, the limited number of large-scale, well-controlled clinical trials restricts the ability to draw definitive conclusions about its efficacy and safety. Addressing these limitations in future research is crucial for advancing our understanding and application of RJ in medicine.

## Future research and perspectives

RJ holds promise as a natural substance with potential health benefits. As a natural material with possible health advantages, RJ holds promise. Several sectors are interested in it because of its high nutrient profile, which includes proteins, vitamins, and minerals. In the future, RJ research may aim to fully exploit its potential in a range of areas, including medical, nutrition, and cosmetics. In order to understand more about its bioactive components and their effects on human health and perhaps develop new supplements or treatments, researchers may investigate them in greater depth. RJ components may be synthesized via biotechnology for a variety of uses. Furthermore, ethical beekeeping practices and sustainable production methods may grow more popular as environmental conservation gains momentum. As scientific understanding grows, there could be a broader range of products incorporating RJ, catering to diverse consumer needs.

## Data availability

Data will be made available on request.

## CRediT authorship contribution statement

**Rajesh Kumar:** Writing – review & editing, Conceptualization. **Ankita Thakur:** Writing – original draft. **Suresh Kumar:** Supervision. **Younis Ahmad Hajam:** Writing – review & editing, Visualization, Supervision, Resources, Conceptualization.

## Declaration of competing interest

The authors declare that they have no known competing financial interests or personal relationships that could have appeared to influence the work reported in this paper.
